# Sesamin inhibits cervical cancer cell proliferation by promoting p53/PTEN-mediated apoptosis

**DOI:** 10.7150/ijms.48955

**Published:** 2020-08-25

**Authors:** Tian-Ni Kuo, Chun-Shiang Lin, Guan-De Li, Cheng-Yi Kuo, Shao-Hsuan Kao

**Affiliations:** 1 Department of Obstetrics and Gynecology, Chi Mei Medical Center, Tainan 710, Taiwan.; 2 Precision Medicine Center, Chung Shan Medical University Hospital, Taichung 402, Taiwan.; 3 Department of Medicine, Medical College, Chung Shan Medical University, Taichung 402, Taiwan.; 4 Department of Biology and Anatomy, National Medical Center, Taipei 114, Taiwan.; 5 Institute of Medicine, Medical College, Chung Shan Medical University, Taichung 402, Taiwan; 6 Clinical Laboratory, Chung Shan Medical University Hospital, Taichung 402, Taiwan

**Keywords:** Sesamin, Cervical cancer cell, p53, PTEN, PUMA, Apoptosis.

## Abstract

**Background:** Sesamin is a major bioactive compound in sesame seeds and has various biological properties, including anti-inflammatory and anticancer activities. Here, we explored whether sesamin activates p53, which is widely inhibited in cervical cancer cells, thereby inducing p53-mediated apoptosis.

**Methods:** Human HeLa and SiHa cervical cancer cells and normal Hs68 dermal cells were used as cell models. Cell proliferation, cell cycle distribution, and apoptosis were evaluated by the CCK-8 assay and flow cytometry using PI/Annexin V staining, respectively. Protein expression and phosphorylation were determined using western blotting. The involvement of p53 in the apoptotic cascade was assessed by a specific inhibitor.

**Results:** Sesamin (75 and 150 μM) clearly inhibited SiHa and HeLa cell proliferation in a dose-dependent fashion, but did not affect the proliferation of Hs68 cells. Meanwhile, sesamin increased the sub-G1 phase ratio and apoptosis, up to approximately 38.5% and 37.8%, respectively. Furthermore, sesamin induced p53 phosphorylation at serine-46 and serine-15 and upregulated the levels of PUMA, Bax, and PTEN, while inhibiting AKT phosphorylation at serine-473. Inhibition of p53 by pifithrin-α significantly reduced the levels of PUMA, Bax, and PTEN but restored AKT phosphorylation in SiHa cells exposed to sesamin. Pifithrin-α also reduced apoptosis and restored the proliferation of HeLa and SiHa cells exposed to sesamin.

**Conclusions:** These findings indicate that sesamin inhibits cervical cancer cell proliferation, and its mechanism may be attributed to the induction of p53/PTEN-mediated apoptosis. This suggests that sesamin might be useful as an adjuvant in promoting anti-cervical cancer treatments.

## Introduction

Cervical cancer is a worldwide life-threatening malignancy in women. The common use of the pap smear for uterine neoplasia screening promotes early diagnosis of uterine cancer and significantly lowers the mortality of patients with uterine cancer 1. However, cervical cancer remains a life-threatening malignancy in women worldwide 2. The death of patients with late-stage cervical cancer is primarily attributed to recurrence and distal metastasis to lymph nodes, lungs, and liver 3-5. Accordingly, the combination of surgical resection, chemotherapy, and radiotherapy is widely used to treat cervical cancer and prevent its recurrence and distal metastasis, and such treatments clearly prolong the overall and progression-free survival of patients with cervical cancer 6, 7.

Although chemotherapy has obvious effects on malignant cancer cells, it also has inevitable cytotoxicity on normal cells and may result in chemoresistance. In the last few years, natural compounds with potential anticancer activity isolated from dietary plants and medicinal herbs have been increasingly and extensively investigated 8. Sesamin is a sesame lignan with a sesamin content of only 0.5-1.2% in sesame seeds. Mounting evidence has shown that sesamin has various properties, including antioxidant and anticancer activities.

*TP53* is an important tumor suppressor gene, and its various mutant forms are widely found in most human tumors. However, in cervical cancer, p53 may be inactivated by binding to viral proteins, rather than through *TP53* mutations. Notably, *TP53* mutations are rarely found in cervical cancer, but more than 90% of cervical cancers are infected with human papillomavirus (HPV) 9. The HPV E6 protein interacts with cellular proteins, E6-AP and p53, and promotes p53 degradation through the ubiquitin-dependent proteolytic system, which may result in p53 dysfunction in HPV-infected cervical tissues 10. Thus, it has been suggested that p53 activation may be a potential anticancer strategy for the treatment of cervical cancer. In this study, we aimed to explore the antiproliferative effects of sesamin on cervical cancer cells, with an emphasis on the p53-mediated apoptotic cascade. Cell proliferation and cell cycle distribution were determined using the CCK-8 assay and flow cytometry, respectively. Protein expression and phosphorylation were assessed using western blotting. The involvement of p53 in the proapoptotic cascade was evaluated using a specific inhibitor.

## Materials and Methods

### Cell culture and reagents

Human cervical cancer cell HeLa and SiHa and dermal fibroblast Hs60 were purchased from Bioresource and Collection and Research Center (Hsinchu 300, Taiwan) and maintained in the culture medium [Dulbecco's modified Eagle's medium (DMEM) containing 2 mM L-glutamine, 1.5 g/L sodium bicarbonate, 0.1 mM non-essential amino acids, 1.0 mM sodium pyruvate, 100 U/mL penicillin, 100 μg/mL streptomycin, and 10% v/v fetal bovine serum (FBS, HyClone, Thermo Fisher, Waltham, MA, USA)]. Cells were maintained grown in a humidified incubator with 5% CO_2_. All the reagents without specific indication were purchased from Sigma-Aldrich (St Louis, MO, USA).

### Cell proliferation assay

Cell viability was assessed by using a Cell Counting Kit-8 (CCK8, Sigma-Aldrich). Briefly, cells were cultured in 96-well plates at an initial density of 3×10^3^ per well, and then incubated with the indicated concentration of sesamin for 24 or 48 h. After the incubation, 10 μL CCK-8 solution was added to each well, and the plate was incubated at 37^o^C for 4 h, then the absorbance at 450 nm was measured using a microplate reader (Molecular Devices, Sunnyvale, CA, USA). The cell viability was presented as mean ± standard deviation at percentage of control.

### Cell cycle distribution analysis

Cells were maintained in the serum-free culture medium for 16 h prior to treatments, to synchronize cell cycle, then treated with sesamin at the indicated concentrations for 24 h in the culture medium. After the treatments, cells were detached by incubation of trypsin, fixed with 70% ethanol, washed with PBS, then reacted with propidium iodide (PI, 20 μg/mL) for 30 minutes in dark. After the reaction, the cells were subjected to cell cycle distribution analysis using a flow cytometer (BD Biosciences, San Jose, CA, USA). Ten thousand cells were counted for cell cycle distribution quantitation using CellQuest Pro software (version 5.1, BD Biosciences).

### Apoptosis assessment by Annexin V/propidium iodide assay

Cells were grown to approximate 80% confluence, treated with DMSO, sesamin, or pifithrin-α following sesamin for 24 h, and the cells were collected for cell apoptosis assessment by using an annexin V-FITC apoptosis detection kit (APOAF, Sigma-Aldrich) according to the manufacturer's instructions. Briefly, cells were washed with cold PBS, and then reacted with Annexin V- FITC and PI (20 μg/mL) in the dark for 20 min. Apoptotic cells were analyzed immediately by BD FACScan flow cytometer (BD Biosciences) with CellQuest pro 5.1. For each sample, the fluorescence of 10,000 cells was gated and counted. The degree of apoptosis was presented as a percentage of the annexin V positive and PI-negative (annexin V+/PI-) cells.

### Western blot

After treated with sesamin at the indicated concentrations for 24 h, the cells were washed with PBS, detached by trypsin incubation, collected, and then lysed with the RIPA buffer containing protease and phosphatase inhibitors for crude protein extraction. The crude proteins were separated using SDS-polyacrylamide gel electrophoresis, then transferred onto an Immobilon P PVDF membrane (Merck Millipore, Billerica, MA, USA). The membranes were blocked with 5% skimmed milk for 1 h, and then incubated with primary antibodies against human p53, phosphorylated p53 at serine 15 [p-p53(S15)], p-p53(S46), PUMA, Bax, PTEN, p-AKT(S473), and β-actin at 4°C for 16 h. After the incubation, the membrane was washed with PBST (PBS containing 0.5% Tween-20), and then incubated with the peroxidase-conjugated secondary antibody (Santa Cruz Technology, Santa Cruz, CA, USA). Antibody complex was detected using chemiluminescence development reagent (GE Healthcare, London, UK), and the resulting chemiluminescent signals were acquired and quantitated using a Luminescent Image Analyzer LAS-4000 mini (GE Healthcare).

### Statistical analysis

Quantitative data were presented as means ± standard errors. Statistical analysis was performed using SigmaPlot (version 11, Systat Software Inc.). One-way analysis of variance (ANOVA) and an unpaired 2-tailed *Student's t*-test was used to determine the significance of differences. *P* value less than 0.05 was considered as significant.

## Results

### Sesamin reduces the proliferation of HeLa and SiHa cells, but not Hs60 cells

**Figure 1:**
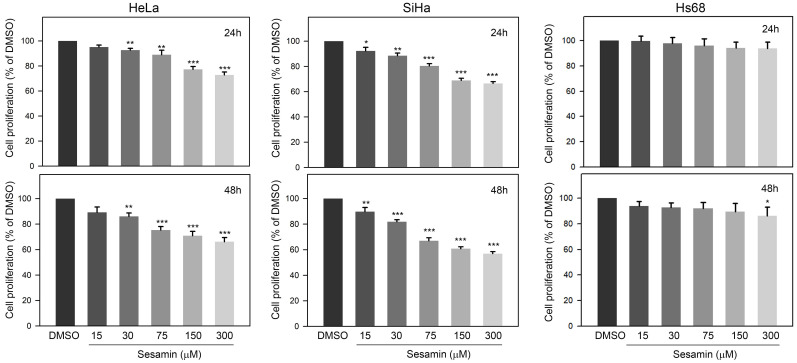
** Effects of sesamin on cell proliferation of human cervical cancer cell HeLa and SiHa and normal dermal fibroblast Hs60.** Cells were treated with sesamin at 15, 30, 75, 150, or 300 μM for 24 h (upper panel) or 48 h (lower panel), and then the cell proliferation was determined using CCK-8 assay. Quantitative data were presented as mean ± SD. Three independent experiments were performed for statistical analysis. *, **, and ***, *P*<0.05, 0.01, and 0.005 as compared to DMSO control.

The effects of sesamin on the proliferation of cervical cancer cells were first investigated. Our observations showed that 24-h sesamin treatments (15 - 300 μM) dose-dependently reduced the proliferation of HeLa and SiHa cells, by up to 74.3±4.2% and 70.5±2.4% of the DMSO control, respectively (Figure 1A, upper panel, *P*<0.001). In addition, 48-h sesamin treatments further inhibited the proliferation of HeLa cell and SiHa cell, up to 69.2±5.2% and 58.9±2.8% of DMSO control, respectively (Figure 1A, lower panel, *P*<0.001). In addition, the effect of sesamin on the proliferation of normal Hs60 fibroblasts was also explored. As shown in Figure 1B, sesamin only mildly decreased the proliferation of Hs60 cells, to 91.3±6.2% of the control at 300 μM (48 h treatment, *P*=0.044 as compared to control). Taken together, these observations indicate that sesamin clearly inhibits the proliferation of cervical cancer cells, but not of normal dermal fibroblasts.

### Sesamin induces sub-G1 phase accumulation and apoptosis of HeLa and SiHa cells

**Figure 2:**
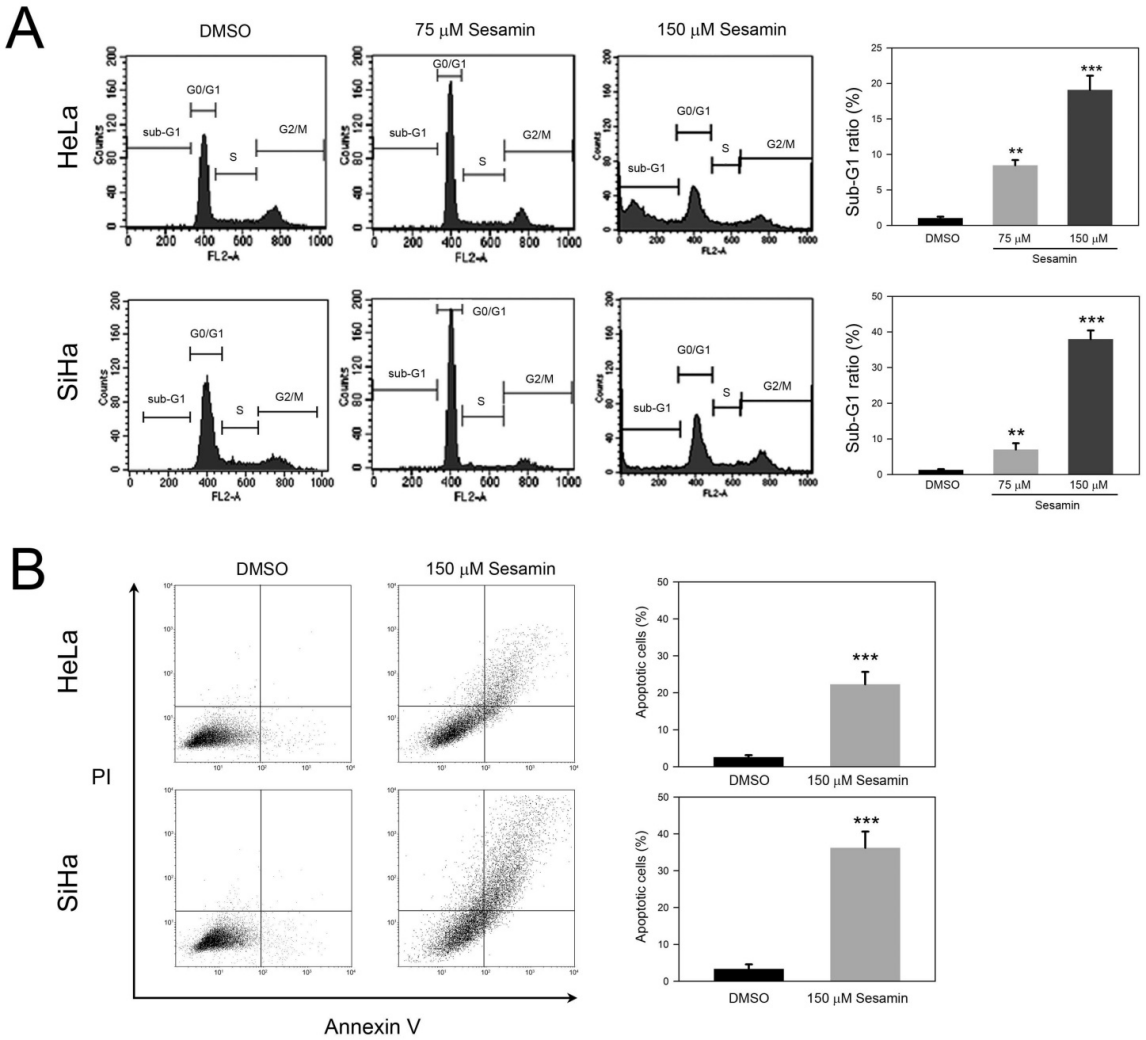
** Sesamin interfered cell cycle progression and induced apoptosis of human cervical cancer cell HeLa and SiHa.** Cells were treated with sesamin at 75 or 150 μM for 24 h, followed by staining with (**A**) PI for cell cycle distribution, or (**B**) PI/Annexin V for cell apoptosis assay, and then analyzed using flow cytometry. Quantitative data were presented as mean ± SD. Three independent experiments were performed for statistical analysis. **, and ***, *P*<0.01, and 0.005 as compared to DMSO control.

To further explore the inhibited cell proliferation in response to sesamin, we next analyzed the effects of sesamin on cell cycle distribution. As shown in Figure 2A, 75 μM sesamin treatment clearly increased G0/G1 ratios, from 66.3±2.7% to 78.4±3.1% (HeLa) and from 64.3±3.4% to 81.3±3.6% (SiHa), respectively (*P*<0.05 as compared to DMSO control). In addition, 75 μM sesamin also increased sub-G1 ratios, to 8.3±1.9% and 7.8±2.3% in HeLa and SiHa cell, respectively (*P*<0.01 as compared to DMSO control). Interestingly, 150 μM sesamin dramatically increased sub-G1 ratios, to 19.1±2.4% and 38.4±2.7% in HeLa and SiHa cells, respectively (*P*<0.005 as compared to DMSO control), while decreasing G0/G1 ratios in both cell types. In addition, annexin V/PI staining was used to demonstrate whether sesamin induced cell apoptosis. As shown in Figure 2B, sesamin clearly increased apoptotic cells, to 21.3±3.4% and 36.5±4.5% in HeLa and SiHa cells, respectively (*P*<0.005 as compared to DMSO control). Taken together, these findings suggest that sesamin induces G0/G1 arrest and eventually results in the apoptosis of cervical cancer cells.

### Sesamin induces a p53-mediated apoptotic cascade in SiHa cells

**Figure 3:**
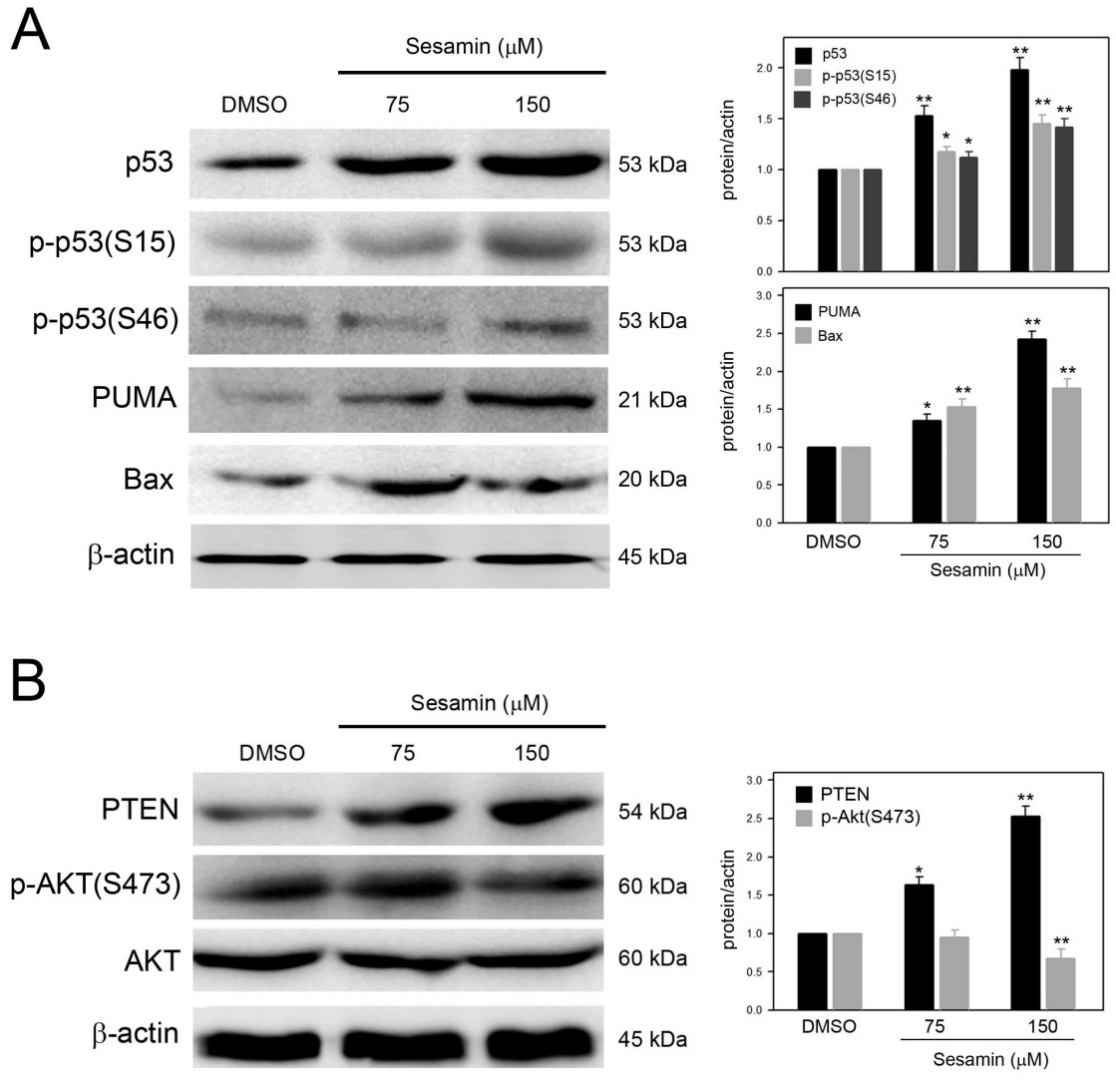
** Sesamin induced p53 activation, upregulated expression of PUMA, Bax, and PTEN, and inhibited AKT activation in SiHa cells.** Cells were treated with sesamin at 75 or 150 μM for 24 h, collected, and then lysed for protein extraction and the subsequent Western blotting. Protein and phosphorylation level were detected using specific antibodies against (A) p53, phosphorylated p53, PUMA, and Bax; and (B) PTEN, AKT, and phosphorylated AKT. Chemiluminescence signal of β-actin was used as internal control. Molecular weights of signals were indicated. Three independent experiments were performed for statistical analysis. *, and **, *P*<0.05, and 0.01 as compared to DMSO control.

Based on the observation that remarkable sub-G1 accumulation was clearly induced in SiHa cells in response to sesamin, the effects of sesamin on the apoptotic cascade in SiHa cells were further investigated. As shown in Figure 3A, sesamin clearly increased p53 protein levels and induced p53 phosphorylation at serine 15 [p-p53(S15)] and serine 46 [p-p53(S46)], in a dose-dependent manner. In addition, PUMA and Bax, the important effectors involved in p53-mediated apoptosis, were also upregulated by sesamin.

PTEN, a tumor suppressor that inhibits AKT activation, can be induced by phosphorylated p53(serine 46). Therefore, whether sesamin induces PTEN expression and subsequently inhibits AKT activation were explored. As shown in Figure 3B, sesamin dose-dependently upregulated PTEN expression, while reducing AKT phosphorylation at serine 473 [p-AKT(S473)] in SiHa cells. Collectively, these results reveal that sesamin upregulates p53 levels and induces p53 activation and the subsequent p53-mediated apoptotic cascade.

### Involvement of p53 in the apoptotic cascade and proliferation inhibition of cervical cancer cells in response to sesamin

**Figure 4:**
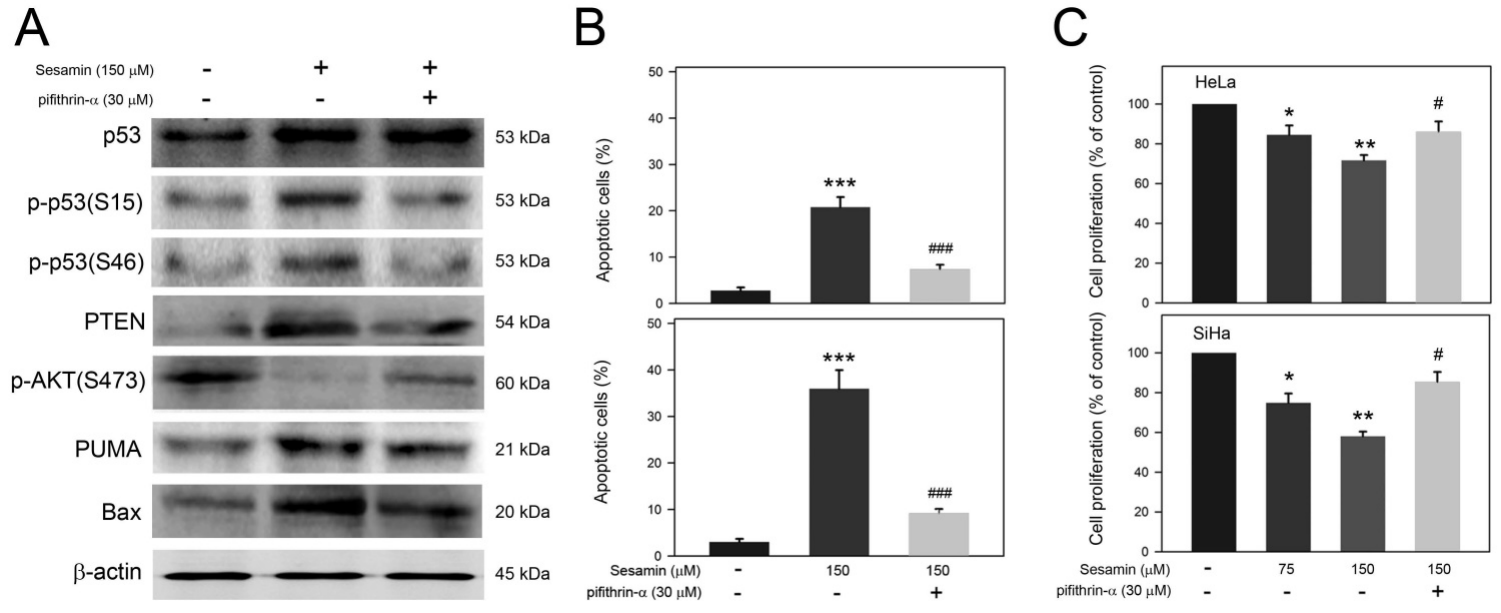
** Involvement of p53 activation in sesamin-induced apoptotic cascade in SiHa cells and sesamin-induced cell apoptosis of HeLa and SiHa cells.** Cells were pretreated without or with pifithrin-a at 30 μM for 2 h, and then treated with sesamin at 150 μM for 24 h. The treated cells were collected, and then (A) lysed for protein extraction and the subsequent Western blotting using specific antibodies as indicated; (B) stained with PI/Annexin V for apoptosis assay; and (C) reacted with CCK-8 for cell proliferation assay. Chemiluminescence signal of β-actin was used as internal control. Molecular weights of signals were indicated. Quantitative data were presented as mean ± SD. Three independent experiments were performed for statistical analysis. *, **, and ***, *P*<0.05, 0.01, and 0.005 as compared to DMSO control. # and ###, *P*<0.05 and 0.005 as compared to sesamin alone.

Based on the observation that sesamin induces a p53-mediated apoptotic cascade and PTEN expression, the involvement of p53 activation in the apoptotic cascade and proliferation inhibition of cervical cancer cells in response to sesamin were next assessed. As shown in Figure 4A, sesamin-induced p53 activation, p-p53(S15) and p-p53(S46), was clearly inhibited by pifithrin-α pretreatment. In addition, pifithrin-α pretreatment also reduced the sesamin-induced expression of PTEN, PUMA, and Bax, but restored AKT activation [p-AKT(S473)] in SiHa cells. In parallel, pifithrin-α pretreatment also reduced the apoptosis of HeLa and SiHa cells in response to sesamin (Figure 4B, P<0.005 as compared to sesamin alone). Moreover, pifithrin-α pretreatment restored the proliferation of HeLa and SiHa cells in the presence of sesamin (Figure 4C, *P*<0.05 compared to sesamin alone). Taken together, these findings show that p53 activation is involved in the sesamin-induced apoptotic cascade, as well as in the sesamin-restrained proliferation of HeLa and SiHa cells.

## Discussion

Epidemiological studies have shown an inverse relationship between the intake of botanical foods and the incidence of several cancers 11-13. Consuming dietary plants benefits cancer prevention, in addition to supplementary treatment with phytochemicals that possess various biological activities, including apoptosis induction, cell cycle regulation, tumor suppressor gene activation, oncogenes inactivation, the suppression of tumor proliferation and progression, and the inhibition of angiogenesis, tumorigenesis, and metastasis. Dou et al. reported that sesamin can lead to apoptosis and inhibit the migratory potential of HeLa cells by inducing ER stress and autophagy 14. Similarly, our results show that sesamin can disrupt cell cycle progression and inhibit the proliferation of human HeLa and SiHa cervical cancer cells by inducing p53 activation, which indicate that sesamin may have potential anticancer activity against human cervical cancer.

p53 is a central tumor suppressor that regulates key genes involved in cell cycle regulation, DNA repair, cell survival, and cell death 15. The p53 cascade governs cell cycle progression by transactivating downstream effectors, such as p21 and p27 16. Moreover, activated p53 also upregulates the expression of PTEN 17, and subsequent formation of the PTEN/p53 complex promotes p53 transcriptional activity, thereby further upregulating the expression of PTEN and p21 and leading to cell cycle arrest 18. In addition, previous studies have reported that sesamin can suppress hepatocellular carcinoma cells via p53/p21-associated STAT3 signaling 19 and non-small cell lung cancer cells via the AKT-mediated p53 pathway 20. Our findings show that sesamin induced G0/G1 arrest at 75 μM and led to remarkable sub-G1 accumulation at 150 μM. In parallel, p53-induced PTEN also suppresses AKT activation. Collectively, these observations reveal that sesamin may induce cell cycle arrest and further apoptotic cascades by upregulating PTEN expression and consequently inhibiting AKT survival signals.

It is estimated that approximately 99.7% of cervical cancers carry integrated HPV DNA in the host genome 21. During HPV infection, two major HPV oncoproteins, E6 and E7, may induce transformation and promote tumorigenesis by binding to p53 and pRb, thereby enhancing the ubiquitin-dependent proteasomal degradation of p53 and dysregulation of cell cycle progression 22, 23. To fully abolish p53 activity, E6 also promotes degradation of the p53 downstream apoptosis inducer Bax 24 and inhibits FADD and pro-caspase 8 25-27. Our findings reveal that sesamin not only activates p53 by phosphorylating serine 15 and serine 46, but also upregulates the levels of PUMA and Bax, which suggests that sesamin might inhibit the binding of E6 to Bax, thereby reducing Bax degradation. In addition, these findings also indicate that the p53-mediated PUMA/Bax axis plays an important role in the anti-proliferative effect of sesamin on cervical cancer cells.

## Conclusions

In conclusion, our results reveal that sesamin possesses anti-proliferative effects on cervical cancer cells, which may mainly be attributed to p53 activation and p53-mediated apoptosis. These findings not only indicate that sesamin could be a dietary-derived compound with potential anti-proliferative activity against cervical cancer cells, but also reveal that p53/Bax might be a potential target for the development of new cervical cancer treatment.

### Funding

This work was supported by the grants from Chung Shan Medical University (CSMU-CMMC-107-04), Chi Mei Medical Center (CMCSMU10705), and from the Ministry of Science and Technology of Taiwan (MOST 108-2320-B-040-026-MY3).
